# Transcatheter Left Atrial Appendage Closure Comes of Age

**DOI:** 10.1016/j.jscai.2023.100592

**Published:** 2023-03-27

**Authors:** Faisal M. Merchant, Mohamad Alkhouli

**Affiliations:** a Section of Cardiac Electrophysiology, Emory University School of Medicine, Atlanta, Georgia; b Department of Cardiology, Mayo Clinic School of Medicine, Rochester, Minnesota

**Keywords:** left atrial appendage, transcatheter left atrial appendage closure

In this issue of *JSCAI*, Saw et al^1^ present the Society for Cardiovascular Angiography & Interventions/Heart Rhythm Society expert consensus statement on transcatheter left atrial appendage closure (LAAC). In many ways, for a procedure such as LAAC, a consensus statement represents a coming of age: It is an opportunity to reflect on where the field has been, to agglomerate existing data, to identify ongoing clinical challenges, and to chart a course moving forward. Undoubtedly, much has evolved in the field of LAAC since commercial approval of the original Watchman 2.5 (Boston Scientific) in 2015. The Watchman has undergone an iterative redesign to the current second-generation Watchman FLX, and a second device, the Amulet (Abbott), was commercially approved in 2021. Numerous other devices are in various stages of development and clinical testing.

So, what does the consensus statement tell us about where LAAC has been? Since commercial approval, there has been rapid uptake of LAAC in the United States, with >100,000 procedures performed at >650 implanting sites registered in the National Cardiovascular Data Registry (NCDR) Left Atrial Appendage Occlusion registry.^2^ From these growth curves, it can be inferred that many new operators and new implanting sites have adopted LAAC in the past several years. Fortunately, despite rapid growth, procedural complication rates remain low and have actually declined. The incidence of pericardial effusion has decreased from >5% in the seminal Watchman trials to <1.5% in the most recent report from the NCDR registry. Importantly, >80% of pericardial effusions can be managed with percutaneous drainage.^3^ In addition, other major complications, such as stroke or death, remain extremely rare, further confirming the excellent safety profile of the procedure.^4^ Maintaining a high level of procedural safety in the setting of rapid expansion requires rigorous standards for physician and institutional training. In this context, the consensus statement offers a useful set of guidelines for initial physician certification and annual procedural volume requirements for maintenance of credentials. Admittedly, the numbers suggested in the consensus statement are empiric because limited data are available on the learning curve with LAAC^5^; however, those numbers are consistent with data on learning curves for comparable procedures, such as left-sided ablation and structural procedures, and they represent a useful set of criteria for the field moving forward.

Additional recommendations are made in the consensus statement for preprocedural, intraprocedural, and postprocedural imaging. Undoubtedly, high-quality imaging has contributed to the excellent safety profile of LAAC, and new operators, in particular, should make liberal use of imaging.^6^ The document deferred the choice of intraprocedural imaging to the discretion of the treating physician. It is likely that the field will see further adoption of intracardiac echo for intraprocedural guidance in the future, especially if the remaining issues surrounding cost and learning curve are resolved.^7^ The document also recommends preprocedural imaging, with a preference for cardiac computed tomography, to guide the choice of device type and size and to guide procedural steps. There has been hope that preprocedural imaging may enhance LAAC efficacy by reducing the incidence of peridevice leak (PDL) and possibly, device-related thrombus (DRT); however, rigorous data on the ability of preprocedural imaging to minimize PDL and DRT remain limited.^6^ Until such data are available, routine preprocedural imaging for experienced operators is likely to remain a matter of individual preference instead of broad consensus.

What does the consensus statement tell us about clinical challenges that persist in the field of LAAC? Most notably, the optimal postprocedural antithrombotic and antiplatelet regimen remains unclear. Indeed, a survey the NCDR registry revealed that the antithrombotic regimen recommended by the instructions for use (IFU) of the original Watchman 2.5 device was uncommonly followed in the United States.^8^ Fortunately, the IFU of current commercial devices (Watchman FLX; Amulet) now allow the option of using dual antiplatelet therapy alone after LAAC in selected patients. The consensus document acknowledges the variability in practice and the limitations of the supporting evidence and leaves physicians with a “general recommendation” to select a postprocedural regimen “according to the studied regimen and IFU for each specific device and tailored to bleeding risks of each patient.” In effect, this recommendation reflects a lack of consensus. A related challenge is the need to define optimal strategies for preventing and treating DRT and PDL. Recent data suggest that both DRT and PDL are associated with an increased risk of stroke/systemic embolism.^2^^,^^9^^,^^10^ Concern is further raised because at the time of postprocedural imaging, typically at 45 to 90 days as recommended in the consensus statement, most patients are still on a relatively intense antithrombotic/antiplatelet regimen. Thrombus may not form on a device until the regimen is tapered; therefore, the reported incidence of DRT (3%-5%) may be an underestimate.^9^ The consensus statement recommends continuing anticoagulation and serially imaging to evaluate for thrombus resolution; however, thrombus has been reported to recur after anticoagulation is stopped, and whether it is safe to ever stop anticoagulation in patients with DRT or PDL remains unclear.^11^ Defining best practices for treatment and prevention of DRT and PDL are challenges that must be addressed by the field soon.

Finally, what does the consensus statement tell us about the course moving forward? Recommendation number 1 in the statement gets to the heart of where LAAC must go moving forward—patient selection. Stroke prevention in atrial fibrillation is fundamentally a balance of competing risks: bleeding risk of anticoagulation, procedural risk of LAAC, and stroke risk if neither anticoagulation nor LAAC are recommended.^12^ Atrial fibrillation is increasingly prevalent in the elderly and those with multimorbidity.^13^ Many of these patients will reach an age or a level of morbidity or frailty where long-term anticoagulation is no longer a safe option. Should all of these patients undergo LAAC? Certainly not. Because there is a greater recognition of the effect of age and frailty on adverse outcomes after LAAC,^14^ there is a greater need to define which patients are most or least likely to benefit from the procedure. The consensus statement makes a novel and important recommendation that patients undergoing LAAC should have a minimum life expectancy of >1 year. This is a tacit recognition of the fact that the benefits of LAAC take time to accrue, and many older and frail patients are unlikely to derive meaningful benefit. Challenges with patient selection are further compounded by the fact that the initial randomized LAAC trials enrolled patients who were eligible for anticoagulation, at least in the short-term. Somewhat paradoxically, after its approval, LAAC is mostly prescribed to patients who are not felt to be good anticoagulation candidates. Unfortunately, there is a paucity of data on LAAC in patients who are poor candidates, or not candidates at all, for anticoagulation; therefore, physicians are placed in the precarious position of extrapolating data to patients who were excluded from the original clinical trials. The consensus statement is right to put patient selection front and center in its recommendations, but as the field of LAAC comes of age, better evidence to support patient selection will be the real sign of maturity.

## Declaration of competing interest

Faisal M. Merchant reported no conflicts of interest. Mohamad Alkhouli is a member of the advisory board for Boston Scientific, Abbott, and Philips and reports institutional research grants from 10.13039/100008497Boston Scientific.

## Funding sources

This research did not receive any specific grant from funding agencies in the public, commercial, or not-for-profit sectors.
